# Using mobile health in primiparous women: effect on awareness, attitude and choice of delivery type, semi-experimental

**DOI:** 10.1186/s12978-024-01785-2

**Published:** 2024-04-10

**Authors:** Fatemeh Moghbeli, Masood Setoodefar, Mohammad Reza Mazaheri Habibi, Zohreh Abbaszadeh, Hanieh Keikhay Moghadam, Sajedeh Salari, Leila Gholamhosseini, Meysam Fallahnezhad, Seyed Ali Fatemi Aghda

**Affiliations:** 1 Department of Health Information Technology, Varastegan Institute for Medical Sciences, Mashhad, Iran; 2 Department of Computer Sciences, School of Engineering, Khayyam University, Mashhad, Iran; 3 Student research committee, Varastegan Institute for Medical Sciences, Mashhad, Iran; 4 Department of Health Information Management, School of Paramedical Sciences, AJA University of Medical Sciences, Tehran, Iran; 5 Department of Health Information Management, School of Health Management and Information Sciences, Iran University of Medical Sciences, Tehran, Iran; 6 Research Center for Health Technology Assessment and Medical Informatics, School of Public Health, Shahid Sadoughi University of Medical Sciences, Yazd, Iran; 7 Student Research Committee, School of Health Management and Information Sciences, Iran University of Medical Sciences, Tehran, Iran

**Keywords:** Education, Telemedicine, Pregnancy, Awareness, Newborn, Primiparous women, Application

## Abstract

**Background:**

One of the reasons for the increase in cesarean section is the lack of knowledge of mothers in choosing the type of delivery. The present study aimed to determine the effect of education through pregnancy application during pregnancy on awareness and attitude and choice of delivery type in primiparous women at Shahid Alavi Specialized Medical Center clinic in Mashhad.

**Methods:**

This study was a semi-experimental type. Seventy primiparous pregnant women who had no restrictions for birth was selected. Sampling was randomized and purpose-based. The data collection tool was a questionnaire. The starting time of the training was considered from the end of the 27th week of pregnancy and continued with regular intervals until the end of the pregnancy. The questionnaires were completed once before the training and once after the training as a pre-test and post-test by the research units. SPSS software (version 26) and MacNemar test and descriptive statistics were used for data analysis.

**Results:**

According to the results of the MacNemar test, a significant statistical difference was seen between women’s knowledge and attitude after the training compared to before (*p*-value < 0.01). Choosing the type of birth was preferred before and after the training. But following the performance of women showed that only 62.86% of them chose natural birth. In fact, before the training, 40% and after 72.86% of women had chosen birth.

**Conclusion:**

Pregnancy education and application during pregnancy is effective in reducing the choice of cesarean section, so this application with the topic of birth and cesarean section has improved the level of attitude and also the positive attitude of pregnant women towards birth. Creation of facilities and promotion of different methods of painless childbirth and training of maternity staff to perform birth is expected.

## Background

The exponential increase in cesarean delivery is seen in both developed and developing countries. Especially in Asia and China, more than 50% of births are performed by cesarean section. In Iran, almost 40% of births in public hospitals and 90% in private hospitals are reported to be by cesarean section [1]. According to the World Health Organization, caesarean section should be between 5 and 15% of all births [2].

The caesarean section, which was first performed to prevent maternal and neonatal complications in certain circumstances, has now seriously increased, which has created a lot of concern for many countries. Cesarean surgery brings many complications for the mother and the newborn, such as: bleeding, infection, increased mortality, premature birth of the baby, breathing problems of the baby, etc. On the other hand, the mother’s disabilities after the operation cause the lack of attention and care of the mother and proper breastfeeding of the baby [3] and impose huge costs on the family [4]. Also, the results of recent studies show that caesarean section has an effect on fertility reduction and postpartum depression [5].

Birth has many advantages compared to cesarean delivery; among the most important of them, the following can be mentioned:

- There are no anesthesia complications in birth, while cesarean section is associated with many risks due to anesthesia.
- The percentage of uterine infection and urinary infection in mothers with normal delivery is lower than that of cesarean section mothers.
- The delivery method plays an important role in regulating the defense system of mother and baby.

Most deliveries are accompanied by pain and discomfort, the intensity of which depends on individual factors, the number and type of delivery, the size and position of the fetus in the uterus, but there are solutions to make it easier for the mother to bear this pain; By having a birth and using pain reduction methods, a pleasant birth experience is created; Today, most of these methods are accessible in Iran.

Several factors are involved in increasing the rate of cesarean section, the type of mother’s choice, old age of the mother, history of previous cesarean section, multiple pregnancy, uncontrollable birth events, fear of pain, stillbirth and uterine rupture are the main factors of this increase [6].

Among the other reasons for the increase in cesarean deliveries are women’s negative attitude towards their previous birth experiences, anxiety and depression, and their difficult social and economic conditions. There are various studies in this field, which have shown the effect of training during pregnancy on the readiness for childbirth [1, 6, 7].

Applications during pregnancy help women to support them in this sensitive and spiritual period, from medical and care tips to devotional advice all in one application. Using pregnancy apps do not guarantee women’s health, but they make things easier and faster for women compared to other methods such as taking notes. The studies conducted show that there are different types of applications related to pregnancy and each of them has its own features and capabilities [8]. Some of these apps are more content oriented and provide users with useful information, while others provide users with a variety of tools ranging from calculators to other tools. Some of these programs also act as a social network that provides a place for dialogue and information exchange [9–14].

**Fig. 1:**
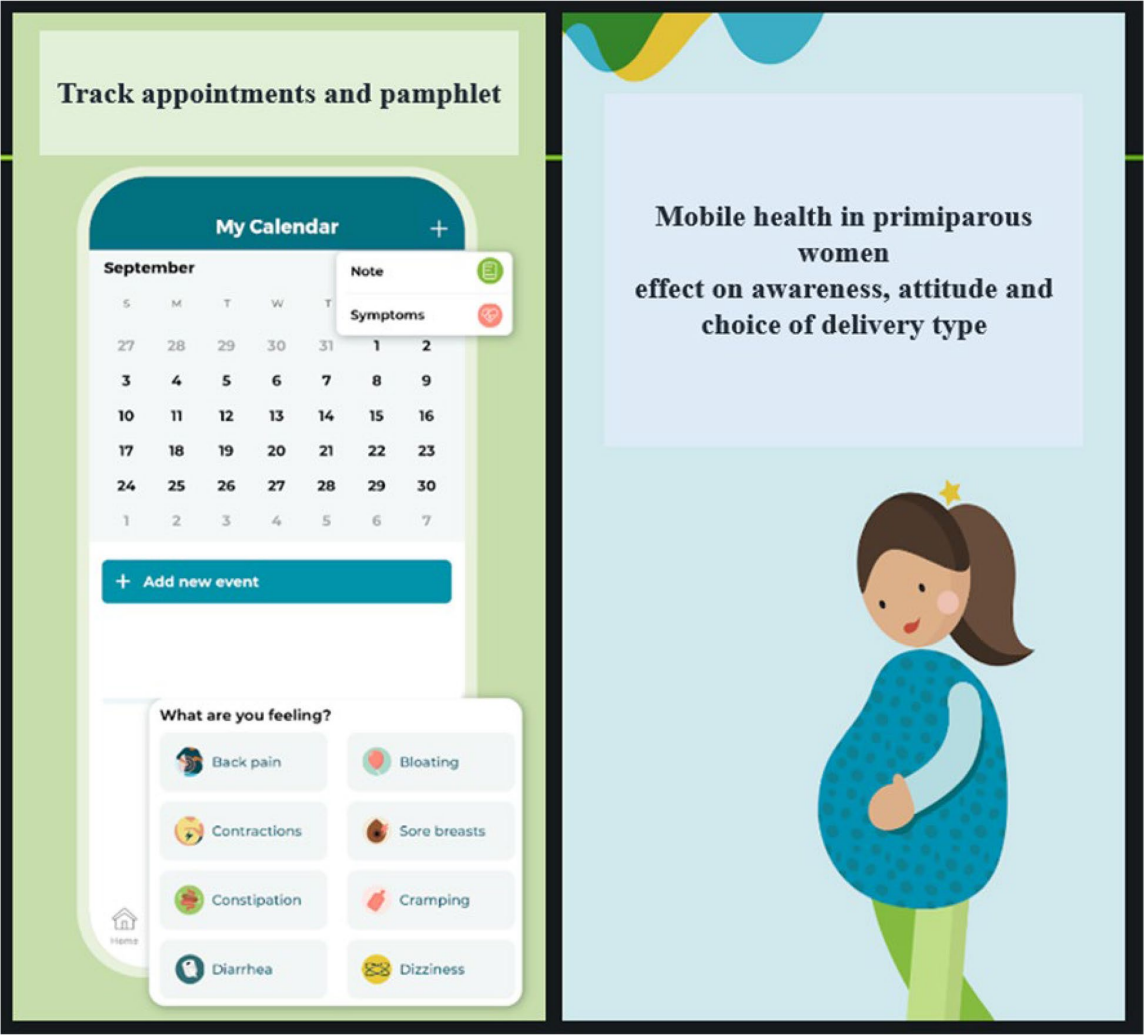
View of the designed application

Based on this study, an application named “*birth or caesarean section*” (Fig. 1) has been designed, which contains content about natural birth, caesarean section, the difference between the two, nutrition during pregnancy, suitable sports for pregnancy, suitable music for this period, and also Provides clips on both birth methods. This application has been approved by two obstetricians and gynecologists. This study shows that the use of other applications in this field may cause problems, because many of them obtain their information from unreliable sources that are not approved by doctors and may cause irreparable complications for the mother and the fetus. This study also used online training, in which the center’s obstetrician explained material to pregnant women that was in line with the content of the app. These classes give women the opportunity to ask their questions about the content of the application or their pregnancy and receive their answers. According to these studies, this research aims to investigate the effect of education through the pregnancy application and online class during pregnancy. Samples have been chosen based the type of delivery in women referring to the clinic of Shahid Alavi Medical Center in Mashhad.

## Methods

This research was a semi-experimental type that was conducted in 2019 at the Shahid Alavi Specialized Medical Center Clinic in Mashhad. The sampling method was multi-stage. First, the file number of the pregnant primiparous people was identified and randomly participated in the research using the lottery method. People who were Iranian and had minimum cycle education, primiparous, pregnant with a single fetus, gestational age over 26 weeks (based on the date of the first day of the last natural period), wanted pregnancy without any history of infertility and no prohibition for birth were included in the research. The number of samples was initially calculated by Cochran’s formula (n$=\frac{\text{n}0}{1+\frac{\text{n}0-1}{\text{N}}}$ ; n_0_: Cochran’s sample size computed using the formula for ideal sample size.; N: the size of the population) to be 80, but before the end of the research, 10 of the samples were excluded from the research due to premature birth (*n* = 70) (inclusion criteria).

The data collection tool included a questionnaire including: demographic characteristics (7 questions), choice of delivery type (1 question), and attitude questions (11 questions). The validity of the questionnaire was determined by reviewing the literature and obtaining opinions from specialists and experts (5 experts in Obstetricians), and its reliability was measured based Cronbach’s alpha was 0.75 (on 30 Pregnant women 27 weeks and above at clinic). To calculate the attitude score, a five-point Likert scale was used, ranging from completely disagree to completely agree and had a score of 1 to 5.

After visiting the clinic of Shahid Alavi Specialized Medical Center of Mashhad, pregnant women who had visited the clinic of Shahid Alavi Specialized Medical Center of Mashhad for pregnancy control were explained about the subject and reason of the research. They entered the study and completed the questionnaire as a pre-test. In this study, an expert Health information technology provider, who worked in the center and considered reliable for pregnant women was used as a trainer. Also, a pregnancy application was installed on the mobile phone of each pregnant woman.

It is a practical application for mothers or women who intend to conceive and give birth. This application provides medical, nutritional, health and educational information on a daily basis according to the day of pregnancy. This program determines each person’s menstrual cycle and calendar personally in order to map their physiological state and increase the chances of pregnancy by predicting the day of ovulation. It is also possible for users to communicate and exchange opinions with each other and to communicate with experts and ask and answer questions with them. This app is designed as a web app with Java programming language, which allows creating a personal account, supports two languages, Farsi and English.

It provides the possibility of communication and questions and answers with support.

At first, the purpose and method of work and the content of educational materials were explained to the teacher. Since the goal and method of the researcher’s work was in line with safe childbirth training classes, the material was familiar to the teacher. The program of designed educational classes started around the end of the 27th week of pregnancy and was held online in the form of a 45-minute session approximately every 10 to 15 days. This program continued until the end of the 38th week. The patients followed until the delivery time by texting message and when they are visiting their doctor.

Also, between classes, pregnant women read and watched the materials and videos specified for them in the application. The educational content taught in the application includes the following:

- When does birth happen?
- Getting to know the stages of birth.
- Who has an easy delivery?
- Types of birth in the hospital.
- Birth with epidural, Birth in water.
- Benefits of birth.
- Birth or caesarean?

These trainings were about 30 h. It was taught about 30 min about the way of using the app. The research team ensure that the research participants used these apps and the app content by tracking them and asking questions. Their use of the app recorded in the users’ and manager’s panel. If the participants did not use the app for the duration/session, they would be excluded from the study (no one found).

After completing the classes (all these women participate in childbirth preparation classes that were held in the centers) and filling out the questionnaires (online), as a post-test, the subjects under the research were followed up to learn about their labor performance and the type of labor. Statistical analysis was first done by SPSS software and using descriptive statistics. Then, the Wilcoxon test was used to compare the knowledge and attitude of the participants before and after the intervention.

## Results

A total of 70 people participated in this research, 41.8% of whom were in the age groups of 21–26 years. The percentage of being employed, the level of education of themselves and their husbands are recorded in Table 1. The average score of awareness and attitude of the research units before the training was 3.09 ± 1.25, which increased after the training and reached 3.15 ± 1.10. This increase was statistically significant (Table 2).

**Table 1:** Frequency distribution of demographic characteristics of mothers and their husbands

Variable	Total percentage
**Education level (bachelor’s degree)**	48.57%
**Wife’s education (senior)**	32.86%
**Job**	58.57%

**Table 2:** Comparison of the average score of the attitude of people before and after training and using the pregnancy application, separating the attitude questions

Questions	Average		*P*
	After	Before	
In cesarean delivery, the patient gives birth to her child after a short sleep.	**2.33**	**3.09**	**0.001**
The choice of cesarean birth is a proof of high social class.	**2.16**	**3.19**	**0.001**
The IQ of a baby born by caesarean section is higher than that of a natural birth.	**2.5**	**3.10**	**0.002**
In my opinion, the main cause of cesarean delivery is fear and lack of awareness.	**3.51**	**2.97**	**0.002**
Natural childbirth increases the emotional connection of the family.	**3.44**	**3.09**	**0.062**
By practicing and getting information about natural childbirth, you can experience it easily.	**3.97**	**2.99**	**0.001**
In my opinion, natural childbirth is the best method of childbirth.	**3.81**	**2.99**	**0.001**
In my opinion, cesarean delivery is the best method of delivery.	**2.21**	**3.06**	**0.001**
In my opinion, by seeing the baby’s face, the fatigue and pain of natural childbirth ends immediately.	**3.61**	**3.09**	**0.003**
In my opinion, consultations during pregnancy can increase excessively	**4.1**	**3.41**	**0.001**
In my opinion, the main reason for choosing cesarean section is pain, which is a useful unpleasant feeling that has the rule of a primary protective mechanism.	**3.11**	**3.04**	**0.691**

According to Table 2, the attitude of the studied subjects regarding each of the items in question, except for two items, had a significant increase 40% of the people under the study before the training had chosen a birth instead of a cesarean delivery, which reached 72.86% after the training. Spouse or friends and acquaintances, choosing the time of delivery, short duration of delivery, etc. According to Table 3, after the training, only 27.14% of people chose cesarean delivery as the preferred delivery. Anyway, despite the preference of birth, only 62.86% of them succeeded in birth.

**Table 3:** Frequency distribution of the type of elective delivery before and after the intervention and McNemar Test

**Variable**	**Number (%) Before intervention**	**Number (%) After intervention**
**Natural childbirth**	28 (40%)	51 (72.86)
**Cesarean**	42 (60%)	19 (27.14)
**Test Statistics**^**a**^		
		before & after
N		70
Exact Sig. (2-tailed)		.001^b^

^a^McNemar Test

^b^Binomial distribution used

**Fig. 2:**
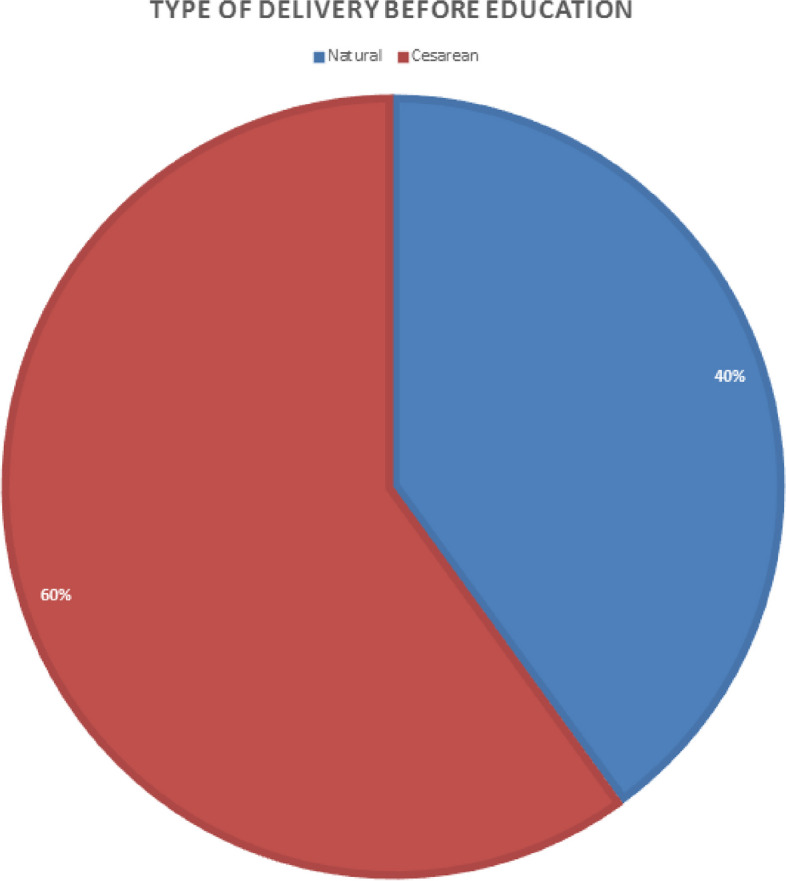
Pie chart related to the type of birth selected before the education of pregnant women

According to this chart (Fig. 2), 40% of the people under study before studying the application and watching online birth classes, and 60% of people chose cesarean section as their chosen delivery method.

**Fig. 3:**
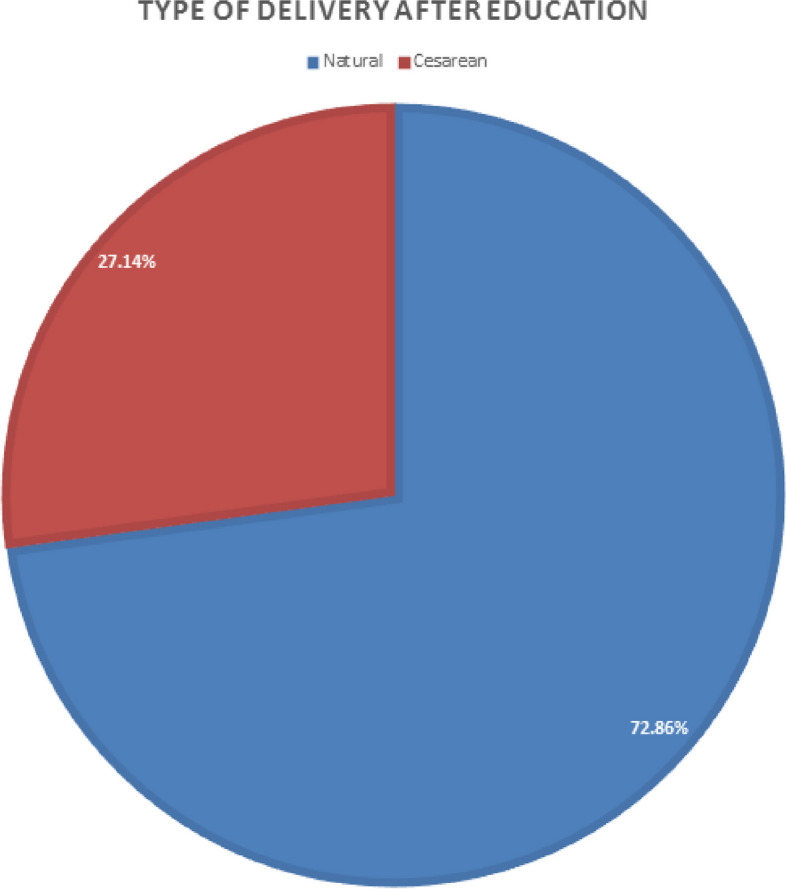
Pie chart related to the type of delivery selected after women’s education

Figure 3 shows the choice of delivery method by pregnant women after reading the application and watching online classes, which 72.86% of pregnant women choose natural delivery due to familiarity with the benefits and Disadvantages of both birth methods and clarification of related issues.

**Fig. 4:**
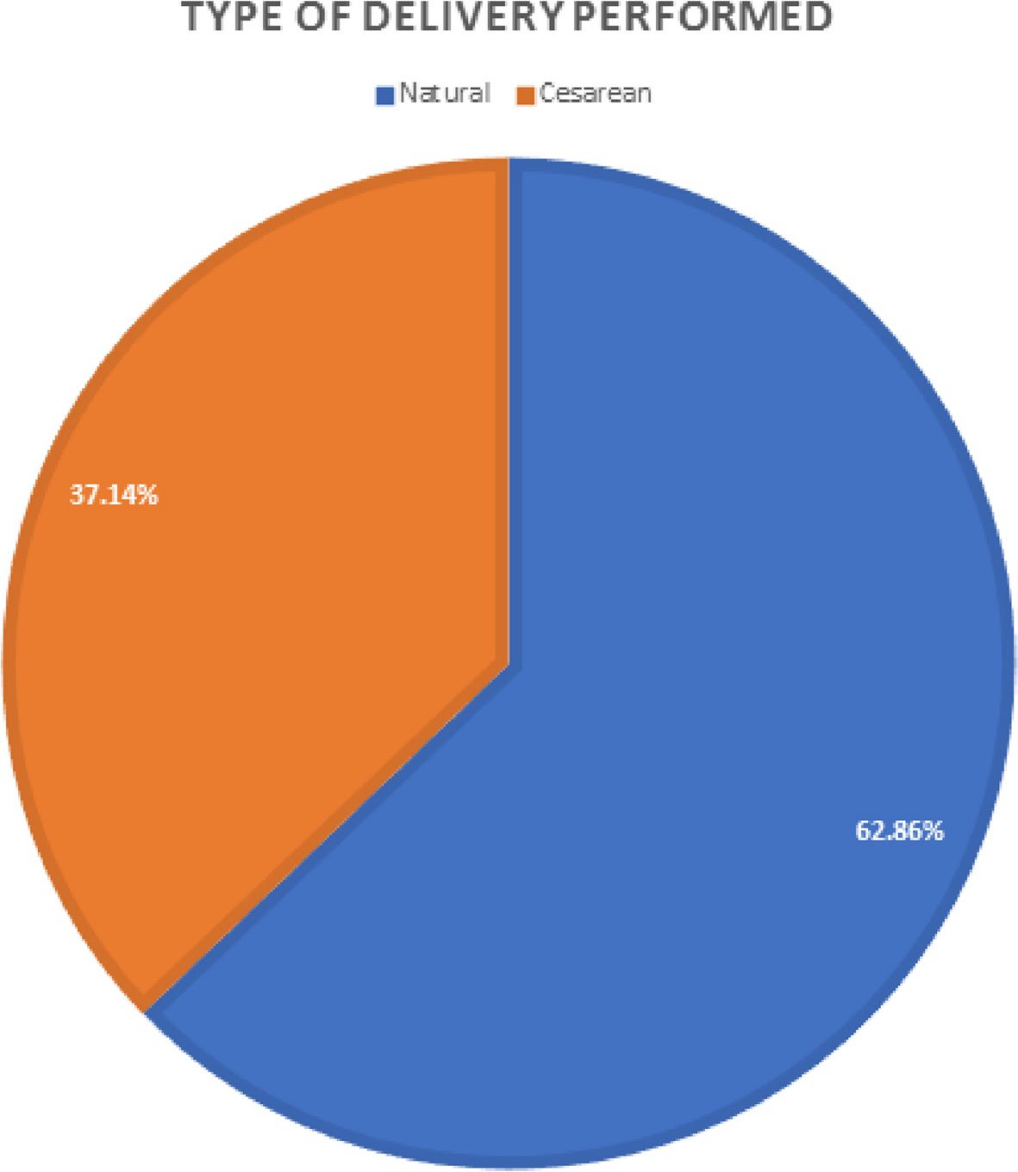
The pie chart related to the type of delivery that took place

Figure 4, shows the results after delivery that only 62.86% of people managed to have a natural delivery and other pregnant women due to issues such as premature delivery, improper condition of the fetus and problems such as Gestational hypertension forced a cesarean section.

## Discussion

Hassanzadeh and et al., conducted a research on the comparison of factors related to the choice of cesarean and birth methods in primiparous mothers and reached the following conclusion: out of 140 cesarean births, 66 births (46.5%) It has no medical advice and one of its most important reasons is the fear of vaginal pain, the safety and health of the fetus. With a 1% increase in awareness about the benefits of birth and the disadvantages of cesarean delivery, the chance of birth increases by 15% and 12%, respectively (*P* < 0.001). Also, with a 1% improvement in socioeconomic status, the chance of choosing a cesarean section increases by 2.45 times. According to the results of this study, increasing awareness about the advantages of birth and the disadvantages of cesarean delivery have a high impact on choosing the natural delivery method. Therefore, considering this case, educational interventions are recommended in the mentioned cases for mothers who intend to become pregnant [15].

Badri and et al., conducted a study on the women’s quality of life after birth and caesarean section and reached the following conclusion: There was no significant relationship between the quality of life of mothers based on the type of delivery, but in the field of physical health. The mothers’ quality of life after birth was significantly higher than the mothers’ quality of life after cesarean delivery. Considering the higher quality of life in the field of physical health in the period after natural delivery, training on emphasizing the effects of this issue on the health of the mother and baby is recommended for specialists and mothers in childbirth preparation classes [16].

In 2017, Mohammad and et al., conducted a research on the effect of specializing educational messages on the choice of the type of delivery in pregnant women and reached the following conclusion: special messages based on awareness, self-efficacy, attitude and perceived social support increase the selection of the method. Birth occurs in pregnant women. Educational programs based on these psychological factors are suggested to guide courtship educational interventions [17].

Ahmadi and et al., conducted a research on the quality of breastfeeding of new mothers in natural delivery and caesarean section in selected military hospitals in Tehran and reached the following conclusion: the quality of breastfeeding in women who gave birth naturally was 114/89 ± 5. 35, which was significantly higher than the quality of breastfeeding in women who gave birth by cesarean section (66.110 ± 36.12) (*P* = 0.045). Since the quality of breastfeeding is higher in birth, therefore, it is recommended to educate and encourage mothers to perform birth [18].

Interventions and complications of childbirth, including emergency cesarean and postpartum hemorrhage, were significantly related to dissatisfaction with childbirth. Such events are common and awareness of these associations may lead to more individualized care of women during childbirth and after [19].

A combination of medical and non-medical factors increases the rate of cesarean deliveries. Nevertheless, our analysis shows that a significant number of cesarean deliveries have taken place in the absence of medical justification. It seems that the factors providing health and treatment services are effective factors in influencing the rate of cesarean delivery in the studied hospitals [20]. 

Most of the women in this study had a positive attitude towards caesarean section if necessary. Lack of formal education, age less than 19 years, and unemployment are associated with the weakness of mothers from cesarean section. Education is necessary to improve the perception of cesarean section as a promoter of child survival, and to cool down the perception that causes the negative result of perinatal birth is especially in the population at risk [21].

The present study was conducted in order to investigate the effect of education during pregnancy on the knowledge and attitude of primiparous women and according to the results, the level of knowledge and attitude of the people under the research improves after the education compared to before, which is similar to the results of other studies [1, 22, 23]. On the other hand, in some studies, education during pregnancy did not have a great effect on raising women’s awareness and, as a result, encouraging them to have a natural birth [24, 25]. The majority of people under the present study, before and after the training, had chosen birth as their desired childbirth method, and similar results were obtained in a research [1]. In another study conducted in this direction, women under training who still had a negative attitude towards birth and had not changed, chose cesarean section (3 to 6 times more than others) as their elective delivery [26].

Fear of childbirth is one of the main reasons why women prefer cesarean section. Fear of natural birth, feeling of security, peace and more control in cesarean section are the reasons of Australian primiparous pregnant women to perform elective cesarean section [27]. Wiklund et al. also believe that primiparous pregnant women suffer from the fear of birth pain and therefore prefer elective cesarean section [28]. In Hong’s study, it was found that Taiwanese primiparous pregnant women consider birth as a They understood the threat to the mother and baby and considered cesarean as a way to eliminate this threat [29]. In the studies conducted in Iran, it has also been stated that women, especially primiparous women, prefer cesarean delivery due to the fear of birth pain, fear of harming the baby and mother, fear of reproductive system disorders, and fear of inappropriate handling in the delivery room. They give [9, 29–31]. Metinnia et al. showed that the severe fear of primiparous pregnant women can be due to insufficient and inappropriate training and their lack of information [32].

Therefore, it can be concluded that the fear of birth is one of the main reasons for preferring cesarean section, especially among young and primiparous Iranian women. Rudsari et al.‘s study showed that proper communication by midwives and doctors with pregnant women and providing childbirth advice to them reduces women’s fear and anxiety to perform birth and encourages them to perform birth [33–35].

In Zamani et al.‘s study, it was found that the most important predictor of choosing the type of delivery is mental norms and modifying women’s attitudes about the benefits and complications of both types of delivery can help increase natural delivery among women [36].

In the design of educational interventions to reduce caesarean section, attitude structures towards choosing the type of delivery and mental norms of primiparous pregnant women should be taken into consideration. Despite the fact that midwives and doctors are influential people in choosing the type of delivery, but various obstacles make the training and counseling done by them ineffective.

Determining the main reasons for choosing a cesarean section and designing a brief intervention and implementing it through people who influence pregnant women (midwives and doctors) can increase the quality and effectiveness of training in health service centers. The content and quality of services provided to pregnant women is a very important and decisive variable in shaping the attitude of pregnant women and the intention to choose the type of delivery. Educational programs, interventions in centers and classes should provide the opportunity for pregnant women to talk about their feelings, concerns and fears. Fear of childbirth is one of the main reasons for preferring cesarean section, especially among young and primiparous women. Therefore, promoting painless childbirth methods and making birth pleasant can be a way to reduce this fear and change the choice of cesarean surgery. Another reason for choosing cesarean is the low awareness of pregnant women about the benefits and side effects of both delivery methods. Training programs that are tailored to the needs and desires of pregnant women can be a way to increase the awareness of pregnant women, and these training programs should be held at a time when the majority of pregnant women can participate [37].

In this research, as a side finding, the delivery performance of the people under study was followed and it was found that although 72.86% had chosen the type of natural delivery after the training, only 62.86% of them had actually experienced a natural delivery. This finding was somewhat unexpected. According to the results of the present research, which shows the effect of education on the preparation for birth, and considering the education provided, the high rate of cesarean in the country can be related to factors other than the mother’s choice, such as unnecessary interventions in the childbirth process.

However, in a study, childbirth preparation method was effective on anxiety during pregnancy and during childbirth and significantly reduced it, and after stopping these trainings, its effect gradually decreased and anxiety increased again [38]. Unlike the present study, which showed a significant relationship between training and changing the attitude of the people under research, in a study, the training provided did not have a significant effect on the attitude of the people [23]. This is probably due to the difference in the educational contents of the emotional domain in the two studies.

## Conclusion

Also, the content of the application that was used in this study was suitable for the needs of pregnant mothers and the creation of the possibility of questions and answers of pregnant mothers in online classes caused the content provided to be specific, and this specialization of the training caused a positive change in the level of awareness, attitude, self-efficacy and perceived social support ultimately increase the choice of natural delivery method in pregnant women.

One of the major limitations of this study was the small sample size, which is necessary to include a larger sample size in future researches. Since the results of this study show the role of childbirth preparation classes and childbirth apps, emphasizing the disadvantages of cesarean birth and the benefits of natural birth, training to restrain different body tension and stretching and breathing movements is effective in reducing unnecessary cesarean sections, in order to achieve the goals of the research, establishing counseling clinics along with providing pregnancy care and also expanding authentic pregnancy applications according to the needs of women.

It is suggested that pregnant women continue to increase the level of awareness and their attitude in choosing the type of birth. Since the reason for increasing the awareness of pregnant women and choosing more birth by them, the observation of many cesarean cases is in conflict with the results obtained, therefore, creating fundamental changes in the field of unnecessary interventions in the delivery department of hospitals, creating facilities and methods Non-medicinal or medicinal methods of painless childbirth, creating suitable conditions for the companionship of the spouse or one of the relatives with the pregnant woman and creating a quiet environment away from any noise of other pregnant women in hospitals are suggested. It is suggested that the macro-programs of countries, which are designed to reduce the rate of cesarean section and promote physiological childbirth, should be followed more seriously.

## Data Availability

The datasets used and/or analysed during the current study are available from the corresponding author on reasonable request.

## References

[1] Tofighi Niaki M, Behmanesh F, Mashmuli F, Azimi H. The Effect Of Prenatal Group Education On Knowledge, Attitude And Selection Of Delivery Type In Primiparous Women. Iran J Med Educ. 2010;10(2):124–30.

[2] Gibbons L, Belizan JM, Lauer JA, Betran AP, Merialdi M, Althabe F (2012). Inequities in the use of cesarean section deliveries in the world. Am J Obstet Gynecol.

[3] Nagibi K, Allameh Z, Montazeri K (2001). Normal delivery vs. cesarean; which one is better.

[4] Sufang G, Padmadas SS, Fengmin Z, Brown JJ, Stones RW (2007). Delivery settings and caesarean section rates in China. Bull World Health Organ.

[5] Mohammadpourasl A, Asgharian P, Rostami F, AZIZI A, Akbari H. Investigating the choice of delivery method type and its related factors in pregnant women in Maragheh. 2009.

[6] Langarizadeh M, Nadjarzadeh A, Maghsoudi B, Fatemi Aghda SA (2023). The nutritional content required to design an educational application for infertile women. BMC Womens Health.

[7] Mazaheri Habibi MR, Moghbeli F, Langarizadeh M, Fatemi Aghda SA (2024). Mobile health apps for pregnant women usability and quality rating scales: a systematic review. BMC Pregnancy Childbirth.

[8] Zolfaqari Z, Ayatollahi H, Ranjbar F, Abasi A (2024). Motivating and inhibiting factors influencing the application of mhealth technology in post-abortion care: a review study. BMC Pregnancy Childbirth.

[9] Langarizadeh M, Sadeghi M, As’habi A, Rahmati P, Sheikhtaheri A (2021). Mobile apps for weight management in children and adolescents; an updated systematic review. Patient Educ Couns.

[10] Baigi SFM, Moradi F, Vasseifard F, Abadi FM, Habibi MRM (2022). The effect of nutrition training on knowledge of students at university of medical sciences. Top Clin Nutr.

[11] Ganjali R, Khoshrounejad F, Mazaheri Habibi MR, Taherzadeh Z, Golmakani R, Mostafavi SM, Eslami S. Effect and features of information technology-based interventions on self-management in adolescent and young adult kidney transplant recipients: a systematic review. Adolesc Health Med Ther. 2019;10:173–90. 10.2147/AHMT.S200801. 10.2147/AHMT.S200801 PMC6800544 31686939

[12] Aalaei S, Amini M, Mazaheri Habibi MR, Shahraki H, Eslami S (2022). A telemonitoring system to support CPAP therapy in patients with obstructive sleep apnea: a participatory approach in analysis, design, and evaluation. BMC Med Inf Decis Mak.

[13] Langarizadeh M, Moghbeli F, Ahmadi S, Langarizadeh MH, Sayadi M, Sarpourian F (2023). Design and evaluation of an educational mobile program for liver transplant patients. BMC Health Serv Res.

[14] Khoshkangin A, Agha Seyyed Esmaeil Amiri FS, Ghaddaripouri K, Noroozi N, Mazaheri Habibi MR. Investigating the role of mobile health in epilepsy management: A systematic review. J Educ Health Promot. 2023;12:304. 10.4103/jehp.jehp_1188_22. 10.4103/jehp.jehp_1188_22 PMC10670869 38023071

[15] Hassanzadeh Talouki H, Faraji Lamoki H, Khatty Dizaabadi F, Yazdani Charati J (2020). Correction to: comparing the factors Associated with selecting normal vaginal delivery or Caesarian Section in Nulliparous Women. J Mazandaran Univ Med Sci.

[16] M KbSOkMBgFM. Assessing the quality of life of women after natural childbirth and cesarean section. International Conference on Health and Health Promotion 2020.

[17] Mohammad K. The effect of specific educational messages on the choice of type of delivery in pregnant women. 2018.

[18] Ahmadi Y, Sharififar ST, Pishgooie SAH, Teymori F, Hoseyni MS, Yari M (2017). Comparison of the quality of Breastfeeding in Postpartum Mothers Undergone Cesarean and vaginal delivery in selected military hospitals of Tehran. Military Caring Sci.

[19] Falk M, Nelson M, Blomberg M (2019). The impact of obstetric interventions and complications on women’s satisfaction with childbirth a population based cohort study including 16,000 women. BMC Pregnancy Childbirth.

[20] Elnakib S, Abdel-Tawab N, Orbay D, Hassanein N (2019). Medical and non-medical reasons for cesarean section delivery in Egypt: a hospital-based retrospective study. BMC Pregnancy Childbirth.

[21] Naa Gandau BB, Nuertey BD, Seneadza NAH, Akaateba D, Azusong E, Yirifere JY (2019). Maternal perceptions about caesarean section deliveries and their role in reducing perinatal and neonatal mortality in the Upper West Region of Ghana; a cross-sectional study. BMC Pregnancy Childbirth.

[22] Ip WY, Chien WT, Chan C (2003). Childbirth expectations of Chinese first-time pregnant women. J Adv Nurs.

[23] Toughyani R, Ramezani MA, Izadi M, Motie Z (2008). The effect of prenatal care group education on pregnant mothers’ knowledge, attitude and practice. Iran J Med Educ.

[24] Kjærgaard H, Wijma K, Dykes AK, Alehagen S (2008). Fear of childbirth in obstetrically low-risk nulliparous women in Sweden and Denmark. J Reproductive Infant Psychol.

[25] Ryding EL, Persson A, Onell C, Kvist L (2003). An evaluation of midwives’ counseling of pregnant women in fear of childbirth. Acta Obstet Gynecol Scand.

[26] Waldenström U, Hildingsson I, Ryding E-L (2006). Antenatal fear of childbirth and its association with subsequent caesarean section and experience of childbirth. BJOG: Int J Obstet Gynecol.

[27] Fenwick J, Staff L, Gamble J, Creedy DK, Bayes S (2010). Why do women request caesarean section in a normal, healthy first pregnancy?. Midwifery.

[28] Wiklund I, Edman G, Ryding EL, Andolf E (2008). Expectation and experiences of childbirth in primiparae with caesarean section. BJOG: Int J Obstet Gynecol.

[29] Bagheri A, Alavi NM, Abbaszadeh F (2013). Iranian obstetricians’ views about the factors that influence pregnant women’s choice of delivery method: a qualitative study. Women Birth.

[30] Faisal I, Matinnia N, Hejar A, Khodakarami Z (2014). Why do primigravidae request caesarean section in a normal pregnancy? A qualitative study in Iran. Midwifery.

[31] Bahadori F, Hakimi S, Heidarzade M. The trend of caesarean delivery in the Islamic Republic of Iran. East Mediterr Health J. 2014;19 Suppl 3:S67–70. 24995763

[32] Matinnia N, Faisal I, Hanafiah Juni M, Herjar AR, Moeini B, Osman ZJ (2015). Fears related to pregnancy and childbirth among primigravidae who requested caesarean versus vaginal delivery in Iran. Matern Child Health J.

[33] Otley H (2011). Fear of childbirth: understanding the causes, impact and treatment. Br J Midwifery.

[34] Langarizadeh M, Fatemi Aghda SA, Nadjarzadeh A (2022). Design and evaluation of a mobile-based nutrition education application for infertile women in Iran. BMC Med Inf Decis Mak.

[35] Nadjarzadeh A, Fallahzadeh A, Abasi A, Poornematy MM, Farahzadi HR, Fatemi Aghda SA (2023). Determining the content and needs assessment a mobile-based self-care program in infertile men. BMC Med Inf Decis Mak.

[36] Zamani-Alavijeh F, Shahry P, Kalhory M, Haghighizadeh MH, Sharifirad GR, Khorsandi M (2020). Identification of factors related to elective cesarean labor: a theory-based study. Daneshvar Med.

[37] Izadi V, Zamanzadeh V, Seyedjavadi M, Mohammadi R, Mazaheri E (2018). Investigation of factors affecting the tendency to choose the type of delivery in pregnant women referring to hospitals of Ardabil Province in 2016. J Family Med Prim Care.

[38] Malekpour Afshar F, Salari P, Azar Pejouh H, Ismaeili H (2005). The effect of education childbirth preparation on anxiety during pregnancy and delivery in primiparous women. J Shahid Sadoughi Univ Med Sci Health Serv.

